# ‘Why is publishing so expensive?’

**DOI:** 10.1242/bio.062557

**Published:** 2026-04-01

**Authors:** Katherine Brown, Daniel Gorelick, Alejandra Clark

**Affiliations:** ^*^ Publishing Director, The Company of Biologists; ^‡^ Editor-in-Chief, Biology Open; ^§^ Managing Editor, Biology Open

For many of us who work in scientific publishing, the title of this Editorial is a question we hear all the time when we're out talking to academics. And it's a perfectly reasonable one. After all, researchers produce the content, for free, and act as peer reviewers to assess whether it's ‘worthy’ of publication – also, in most cases, for free. Biology Open (BiO) is a notable exception to this latter principle since we are financially compensating reviewers for their work (Gorelick and Clark, 2025), but we are one of only a very few journals to do so. In general, though, if most of the work is done by academics and ‘all’ the journal does is put the final version online, how can that possibly cost US$2500 [the current Article Processing Charge (APC) for BiO]? In this Editorial, we (the Editor-in-Chief and Managing Editor of BiO and the Publishing Director of The Company of Biologists, our not-for-profit publisher) provide details and context on BiO's finances, and hope to dispel some of the myths around the economics of publishing.

For those of you who want the take-home message up-front, the two key points we want to make here are firstly that quality editorial assessment and publishing involves a lot of people (meaning our largest costs are staff salaries and Academic Editor and reviewer stipends), and secondly that publishers have had to invest heavily in technologies to support professional and trustworthy online publishing – there's a lot more to it than just posting an article on a website. But before we get into the details, here's how a typical conversation with a researcher might go when talk turns to money:

## Why should we use hard-earned and often taxpayer-funded grant money to enrich publishers when free platforms such as bioRxiv are available?

Preprint servers are fantastic, but they're not journals. Preprints don't incur the same costs because they don't go through the kind of rigorous screening that happens at a journal such as BiO – both through peer review and through the technical and integrity checks we perform, which help to ensure that readers can and will trust your work. And thanks to our ‘Fast & Fair’ initiative, all this happens on an accelerated timescale. Not only that, but by publishing in an Open Access (OA) community-centric journal such as BiO, you can maximise the chances of your paper being seen and used by your colleagues. Our post-acceptance processes (outlined in more detail below) will ensure it is in the best possible shape to be read by the community, it will get indexed in PubMed and other services, it will land in people's inboxes and on their social media feeds and, in some cases, it will get featured through an author interview. As discussed below, all these things take time and money and are key services that publishers can provide but preprint servers can't.

## But surely publishing should be much cheaper now journals are online-only?

Professional online publishing isn't as cheap as it might initially seem. BiO has always been an online-only journal, but the actual process of printing our sister journals Development, Journal of Cell Science and Journal of Experimental Biology (which we did until the end of 2024) was a very minor outgoing in the context of all costs incurred. And given that 25% of full-text views to BiO articles are to the PDF version rather than the HTML, it's clear that readers still want to read in PDF format, meaning we still need to typeset each article. Not only that but we, in collaboration with our online hosting partners Silverchair, need to make sure that articles are preserved in perpetuity, meet modern accessibility requirements and that the online reading experience provides the kind of functionality that readers expect. It may not all be visible, but a lot of work goes into maintaining and improving the website.

## What about page charges and length limits? They can't be relevant in an online world, can they?

Firstly, none of The Company of Biologists' journals have page charges, although some journals at other publishers still do. But article length is still relevant – we are charged for typesetting by the page, and a longer paper typically takes longer (and therefore costs more) to copyedit and proofread. That said, the reason we believe length limits still have a place is not because of cost but rather because we want to encourage conciseness for the reader's benefit.

## Why is there such variation in the APCs set by different publishers?

APCs at well-established journals like BiO can vary from a few hundred dollars to over $10,000. There are many reasons for this variability, ranging from the economic model of the publisher (for-profit versus not-for-profit) and the scale of the operation through to the staffing model of the journal (professional versus academic editors; in-house versus outsourced production processes). One factor that's often under-appreciated is the selectivity of the journal, i.e. what percentage of papers received are eventually published. An OA journal that publishes the majority of its submissions receives income for most of the time and money expended in assessing those manuscripts. By contrast, a highly selective journal spends a lot of time processing papers that won't get accepted and therefore bring in no money. Many publishers have tried to get around this problem by providing trickle-down cascades to keep papers (and the income they represent) within the same publishing house, but at an individual journal level, the more selective you are, the more you have to charge to recoup your costs. Of course, you can argue about the value of such selectivity, but that's a whole separate conversation…

## Financial transparency in an OA world

In years gone by, the traditional subscription-based publishing model typically provided healthy revenues for publishers big and small. For The Company of Biologists (which publishes four journals in addition to BiO: Development, Journal of Cell Science, Journal of Experimental Biology and Disease Models & Mechanisms), we ensured this surplus went back to the community in the form of charitable activities while also maintaining an investment pot to provide financial security to the organisation (see Box 1). But we are now in a very different world. Over the past two decades, the laudable push for OA publishing has led to an almost bewildering array of publishing options, business models and author-facing charges. Meanwhile, as discussed below, publishing has become a more complex (and costly) process. Given that revenues from OA streams are typically smaller than those from subscriptions, finances at small publishers like us are now significantly tighter: we now operate our journals at a close to break-even budget, and our investment pot is the primary source of our charitable funds.

Box 1. The Company of Biologists' finances and charitable activities As a UK-registered charity, The Company of Biologists exists to benefit the scientific community and not to profit shareholders. Our charitable activities include the following:

- organising and hosting scientific Workshops and Meetings for the biological community
- providing scientific meeting grants to help defray the costs of organising conferences and support scientific interactions
- funding early career researchers undertaking collaborative visits to other labs
- providing travel grants for researchers attending conferences and training courses
- block grants to three UK-based societies (the British Society for Developmental Biology, the British Society for Cell Biology and the Society for Experimental Biology) to support their activities.

We know from community feedback how vital this support is to researchers across the globe.

In the past, funds for these activities came from the surplus made by our publishing programme. However, our publishing costs have grown significantly in recent years (by 22% in real terms – after accounting for inflation – between 2015 and 2024). We have worked hard to avoid passing these costs on to our community and, as noted elsewhere, OA-based revenues are typically lower than those from subscriptions. Given these challenges, our income has decreased by 10% in real terms over the same period despite continued growth in R&P participation powered by successful relationships with libraries. Consequently, while in 2015 our surplus was able to fund all our charitable activities, in 2024 it represented less than 40% of our charitable spend, with the rest coming from the growth in our investments.

We remain committed to supporting the scientific community well into the future and are fortunate – thanks to prudent investments over the past 100 years – to be in a position to do so despite the challenging financial circumstances that small publishers are facing.

**Fig. 1.**
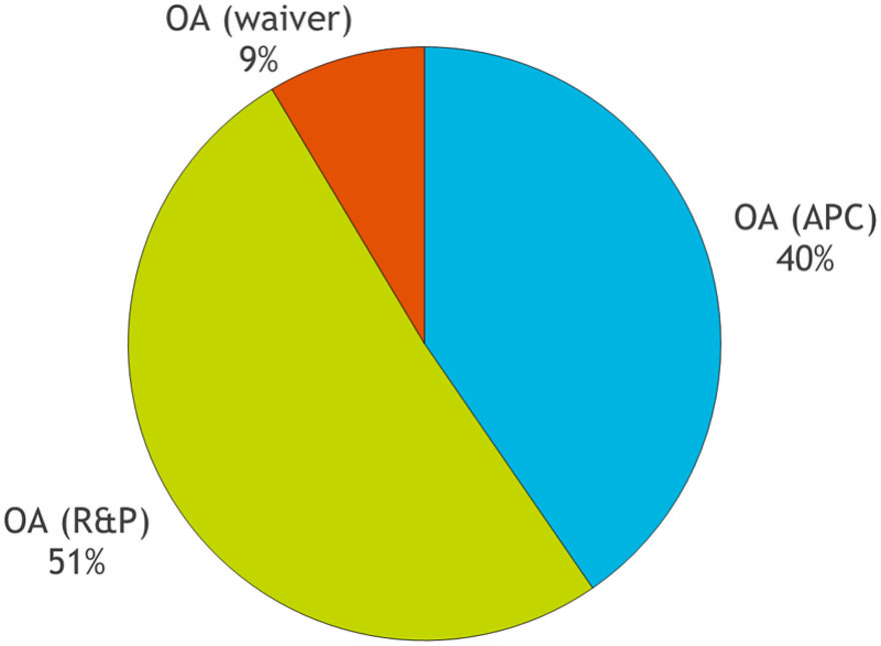
**Breakdown of research papers published in 2025 by OA type: individual author payments (APCs), institutional agreements (R&P) or waived fees.** Automatic waivers are granted to authors from lower and lower-middle income countries, and can also be requested by authors from other regions.

Academics also tend to be much more aware of the money side of things these days because they are often required to pay publishing fees from grant funds. In this context, publishers have a responsibility to be more transparent about where that money goes. So, let's start with the revenue coming in. Until recently, BiO's sole income stream was the APC payments made by authors. Over the past few years, however, we have begun to include BiO (and our other fully-OA journal, Disease Models & Mechanisms) in our Read & Publish (R&P) agreements with librarians. These allow fee-free OA publishing for authors of participating institutions (see https://www.biologists.com/library-hub/read-publish/) and many institutions have chosen to opt in to our five-journal agreement. As seen in Fig. 1, many of BiO's papers are now published through the R&P route. Note also that we strongly believe that no author should be deterred from publishing with us due to financial considerations; we therefore offer a generous waiver scheme for authors unable to cover the costs of the APC or benefit from an R&P agreement.

Given BiO's history as an APC-based OA journal, and because the APC level also feeds into R&P pricing (becoming an increasingly significant contributor as the proportion of OA articles grows across our portfolio), we aim to set the APC at a level that begins to cover our costs per article, ensuring financial viability of the journal.

### What about the costs?

It is important to note that as a small not-for-profit publisher of only five journals, we do not benefit from any of the economies of scale that can be achieved by larger organisations. We publish our journals ourselves (rather than partnering with a larger publisher, as many society and non-profit journals do) because this gives us full control of how we operate and the quality of our product. These are significant benefits to going it alone, but the financial challenges are exacerbated by not being part of a large stable of journals with common resources and opportunities for collaboration. We also note that BiO's costs are significantly lower than our other journals, partly because BiO does not have a significant ‘front section’ (review-type articles) or the staff associated with producing this.

**Fig. 2.**
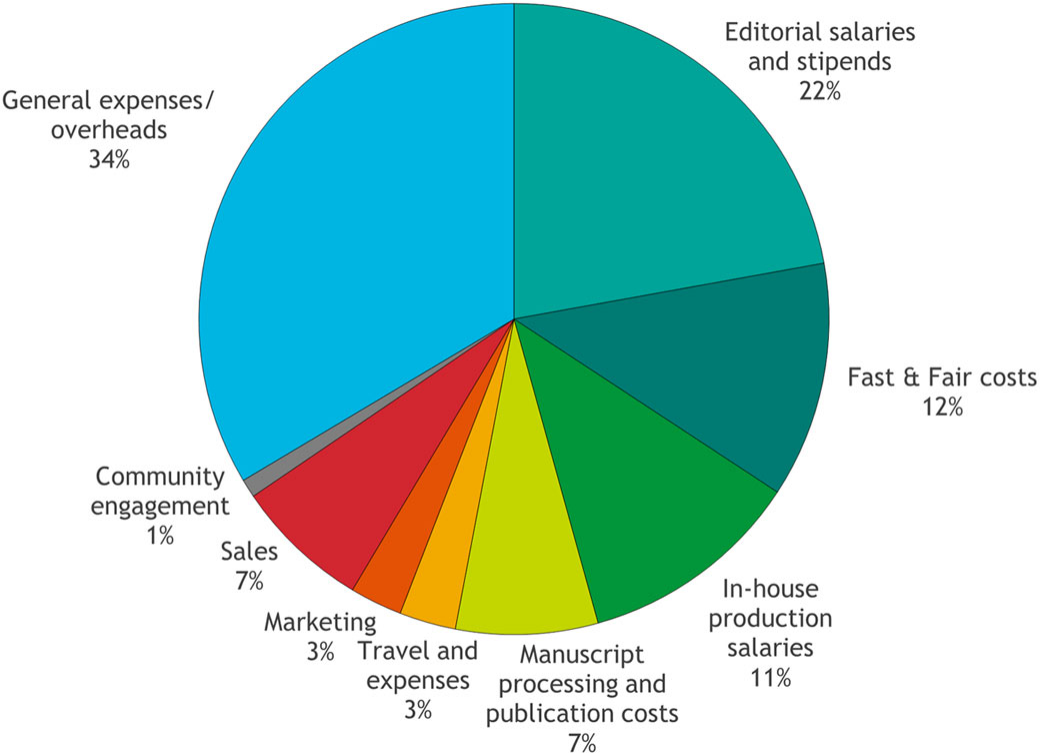
**Breakdown of costs for BiO in 2025.** Manuscript processing and publication costs include services from our typesetting service provider, manuscript submission system and online hosting platform. Travel and expenses are costs incurred in conference attendance by our Managing Editor and any Academic Editor travel on behalf of the journal. Community engagement includes costs of running and staffing our community site, preLights.

Our biggest direct cost area is in salaries and stipends (Fig. 2). The BiO in-house team consists of three people (2.4 full time equivalents) – the Managing Editor, a Production Editor and an Editorial Administrator. Producing the journal also requires work from our Technical Operations department, including graphics and proofreading teams. Without these committed staff members, manuscripts wouldn't move through the assessment and peer review process, and accepted papers wouldn't get copyedited (checked and corrected for accuracy, clarity and compliance with key journal policies) or scrutinised in-house for integrity issues. In short, BiO just wouldn't get published. Equally important are our team of Academic Editors, who are compensated financially for their time and dedication to the journal – reading submissions, selecting peer reviewers, making decisions on which articles to publish, supporting authors through the publication process and contributing to the overall strategic directions of the journal. For BiO, costs have recently increased due to the implementation of our ‘Fast & Fair’ initiative, which adds £440 in direct costs for each manuscript undergoing peer review. Costs that can be directly attributed to Fast & Fair (reviewer payments and software costs associated with managing our reviewer pool) are specifically shown in Fig. 2 and make up 12% of total per-article costs.

It's all very well producing a journal and papers to go in it, but we also need to make sure that people can find and read them, and this is where our Sales and Marketing department comes in. As mentioned earlier, The Company of Biologists has been very successful in growing its OA content through R&P agreements, but this has required significant investment in our Sales team. Meanwhile, we rely on our Marketing staff to help us promote the journal and its papers, to reach new readers and authors and to gather feedback on what we do well and where we can improve. Finally, of course, there are all the general expenses and overheads – like any organisation, we need to manage and develop the business and its people, we need finance and HR support, and we need to keep the lights on and the laptops running.

As well as our people and facilities, we are also reliant on a host of technology partners and platforms – from our manuscript submission system to our typesetting service provider to our online hosting platform. And it's not enough just to put up a PDF: as noted above, we need to keep on top of emerging standards and evolving expectations from readers and librarians, providing, amongst other things, fully accessible XML, play-in-place movies, archiving, indexing and appropriate linking to relevant databases. Increasingly, we've also had to invest in software solutions and staff time to detect and address publication integrity issues, including plagiarism, inappropriate image manipulation and papermill submissions. The age of AI will only exacerbate these issues.

## Quality publishing takes a team

Publishing, done well, is an expensive business. For every paper you read in BiO, at least four different in-house staff – an Editorial Administrator, the Managing Editor, a member of our graphics team and a Production Editor have been involved in its journey from submission to publication, as have at least one Academic Editor, two peer reviewers (the majority of whom are now paid) and the (outsourced) typesetting team. And across the whole organisation, we employ 62 full- or part-time staff whose roles support our publishing operations (not counting those solely dedicated to our charitable activities). We are proud to be a people-centric organisation, and while we're actively assessing options to streamline processes and reduce costs, the quality you expect from BiO will continue to be at the forefront of our thinking.
